# Antibiotic-Loaded Cement in Orthopedic Surgery: A Review

**DOI:** 10.5402/2011/290851

**Published:** 2011-08-07

**Authors:** Alessandro Bistolfi, Giuseppe Massazza, Enrica Verné, Alessandro Massè, Davide Deledda, Sara Ferraris, Marta Miola, Fabrizio Galetto, Maurizio Crova

**Affiliations:** ^1^Department of Orthopedics and Traumatology, AO CTO Hospital, Turin, Italy; ^2^c/o AO CTO/M. Adelaide, Via Zuretti 29, 10126 Torino, Italy; ^3^University of the Studies of Turin, Turin, Italy; ^4^Materials Science and Chemical Engineering Department, Polytechnic of Turin, C.so Duca degli Abruzzi, 24-10129 Turin, Italy; ^5^Department of Clinical and Biological Sciences, c/o S. Luigi Hospital, Regione Gonzole, 10-10043 Orbassano, Italy

## Abstract

Infections in orthopaedic surgery are a serious issue. Antibiotic-loaded bone cement was developed for the treatment of infected joint arthroplasties and for prophylaxes in total joint replacement in selected cases. Despite the widespread use of the antibiotic-loaded bone cement in orthopedics, many issues are still unclear or controversial: bacterial adhesion and antibiotic resistance, modification of mechanical properties which follows the addition of the antibiotic, factors influencing the release of the antibiotic from the cement and the role of the surface, the method for mixing the cement and the antibiotic, the choice and the effectiveness of the antibiotic, the combination of two or more antibiotics, and the toxicity. This review discusses all these topics, focusing on properties, merits, and defects of the antibiotic loaded cement. The final objective is to provide the orthopaedic surgeons clear and concise information for the correct choice of cement in their clinical practice.

## 1. Introduction

The purpose of this review is to analyze the main issues of antibiotic loaded bone cements and to comment their basic properties, main characteristics, merits, and defects. The final goal is to provide the orthopaedic surgeons of clear and concise information for a correct choice of antibiotic loaded cement in the clinical practice.

### 1.1. Biomaterials, Infections, and Orthopedics

The presence of biomaterials in orthopedic surgery involves a high risk of developing deep infections [1]. One of the main factors is the phenomenon of the adhesion of the bacteria to the biomaterials and the production of a biofilm from the bacterial strains [2–5]. In fact, it was demonstrated that bacteria have the ability to bind to the surface of biomaterials, due to specific physical and chemical properties [2, 6, 7]. Infections around joint arthroplasties are among the most difficult to manage and to heal. Until decades ago, the antibiotics available for the prevention and the treatment of the orthopedic infections were only a few and these antibiotics could have been ineffective against certain bacteria like staphylococci and gram-negative. With the spread of the prosthetic joint replacement in the seventies, the problem increased [1, 8–10]. Actually, the mainstay of treatment of an infected joint prosthesis is based on the removing of the implant and on the accurate toiletries around the surrounding necrotic soft and bone tissue, either in on- or two- stage technique [11–13].

The positioning into the surgical site of cement loaded with antibiotics may be useful to maintain at local level a high concentration of drug, which could not be reached by the venous administering without general complications and toxicity [14, 15]. Nevertheless, the real effectiveness of the antibiotic loaded cement is currently under debate [16]. After the surgical treatment and cleaning, the systemic administering of antibiotics for prolonged time is anyway mandatory, being the toilet alone not enough. After years of scientific debate, there still are many doubts and conflicting opinions on several aspects of the use of antibiotic cement: the method of preparation, the choice of the antibiotic, the effective release and diffusion of the antibiotic in the surrounding tissues, and the mechanical properties of the antibiotic loaded cement.

## 2. Cement Development and Joint Infections

 The subject of this review, the so called bone cement is a polymer-based material composed of poly-methyl-methacrylate (PMMA) or copolymers, is a polymeric material commonly used for the fixation of the joint implants to the bone. 

 In recent years, thanks to the improved surgical techniques, to the adoption of stringent and efficient antiseptic pre-operative and intra-operative procedures and, above all, thanks to the optimization of the peri-operative systemic antibiotic prophylaxis a significant reduction in the number of deep infections and subsequent revisions occurred [17–20]. It was estimated that the rate of the infections was reduced from 5–10% to approximately 1-2% during the last twenty years. Among the many procedures to fight periprosthetic infection, the use of antibiotic enriched bone cement is widely used [21], particularly in case of revision and septic failure of the arthroplasties. Over 30 years ago, Bulchoz and Engelbrecht reported that penicillin, erythromycin, gentamicyn introduced into the cement used to stabilize the hip, spread into the surrounding tissues for months, bringing as a result a prolonged local concentration of antibiotic [22]. After these findings, the interest in the application of cement impregnated with antibiotic in the treatment of osteomyelitis grew. In 1979, as an alternative to the introduction of large deposits of antibiotic cement in the site of chronic osteomyelitis, Klemm introduced gentamicyn in cement beads and used them as temporary filler for the gap that was created after the removal of necrotic tissue. The cement impregnated with antibiotic (ALBC: antibiotic loaded bone cement), since the late 90ties, was increasingly used for the prevention of the arthroplasty-related infections. Also, over the time, this choice has undergone significant changes and improvements concerning the chemical formulation, the techniques of preparation and clinical applications [22].

## 3. Bacterial Adhesion and Antibiotic Resistance

The addition of antibiotics to the cement must be considered as a support strategy in preventing the onset of infections and not the solution: the key point is still represented by the sterility in the operating room and by the antiseptic surgical procedures. Nevertheless, any procedure which can potentially reduce the adhesion and the bacterial colonization is welcome in orthopedics. Hypotheses were formulated about the effectiveness of the addition of antibiotics in terms of reduction of the bacterial biofilm on the different types of cement and it appeared as a multi-factorial process, not related only to the kinetics of release of the antibiotic. Some sustained that the production from the bacteria of a kind of glycocalyx (extracellular structure that covers the external surface of tissues with a “sheath” that is found mainly in epithelia), which adheres to the biomaterial, causes physiological changes on bacteria themselves and confers antibiotic resistance [16, 23]. Others proposed that the main factor can be the hydrophobicity of the implanted material, the electrostatic interactions and/or the roughness of the surface [24, 25]. Emerging evidences showed that the bacterial adhesion to a biomaterial is the result of a development of the antibiotic resistance [22]. It was hypothesized that the bacterial growth is privileged on certain biomaterials: for example, coagulase negative staphylococci would prefer to join the bone cement, while *S. Aureus* would show preferential adhesion to metallic biomaterials [26]. Prolonged exposure to antibiotic at a dose concentration below the inhibitory one, allows the development of mutational resistance in bacteria. Therefore, the wide clinical use of ALBC with preventive purposes must be carefully considered [16, 22]. The use of cement added with gentamicin for first implants was associated with the development of coagulase-negative staphylococci resistant to this drug [27]. Also, bacterial strains resistant to gentamicin were found in the 88% of the cases of infection in arthroplasty where cement was loaded with antibiotic, compared to the 16% found after those where the common cement was used [28, 29]. Another important factor for bacteria adhesion is the roughness of the surface: in general the higher the roughness, the higher the adhesion and the PMMA is characterized by a rough surface [11].

## 4. The Use of Antibiotic-Loaded Bone Cement: General Principles

The PMMA enters the operating room packaged in monomer (liquid) and polymer (powder) separately. At the time of the preparation, when mixed, it becomes a viscous material paste, which solidifies in few minutes by an exothermic reaction. It acts as a fixation between the prosthetic components and the cancellous bone. During the mixing, pores of different sizes are produced as consequence of the chemical reaction and volume variations. These microholes may represent the start point of cracks and thus can be responsible of the premature failure of the cement. To avoid the formation of these pores, it is possible to prepare the cement under vacuum conditions. Nevertheless, the preparation under vacuum leads to a greater reduction in volume during the polymerization, thus resulting in higher shrinkage and worse adhesion on the bone-prosthesis interface compared to the nonvacuum mixed cement. It was demonstrated that among the compounds prepared in the operating room, those made under vacuum conditions present improved mechanical properties [30–32]. However, it is not the purpose of this work to discuss the method of preparation of cement and the relative advantages or disadvantages of the vacuum preparation on the biomechanical properties. 

Some authors do not recommend the use of antibiotic cement in primary arthroplasty [30], first of all for the reduction of mechanical properties and secondly because its spread use might lead to the selection of antibiotic resistant bacteria. The use of the antibiotic loaded cement in primary implants is indicated in patients with hag surgical risk, in elder patients, in patients with general health problems like immuno-depression, diabetes, history of previous prosthetic and periprosthetic infections, and particular diseases such as rheumatoid arthritis and SLE, or in conditions of malnutrition [33, 34]. In contrast, the use of the antibiotic loaded cement is recommended by most authors for joint arthroplasty revisions, which are at higher risk of infection compared with the first implants [33, 34]. The use of the antibiotic-loaded cement is particularly indicated for septical revisions [30]. The revision of an infected joint arthroplasty can be performed in a single surgical operation (one-stage), where the removal of the implant and of the infected and necrotic tissue is followed by an accurate cleaning of the area and by the implant of the new prosthesis. On the contrary, the revision can be performed with the so-called “two-stage” technique, where the first step is the implant removal and the surgical toilette, then a temporary spacer is implanted, and finally the new prosthesis is implanted at a distance of 6–8 weeks after surgery with a new operation [12, 13, 35]. During the period of time between the two surgeries, the area of the joint cleaned up can be left empty or most commonly is filled with a spacer. Antibiotic loaded cement is the most used spacer, due to its plasticity and to the capability to release the antibiotic *in situ* [27, 30, 36]. In this case a therapeutic local effect is added to the primary function of the spacer: to avoid the retraction of the tissues and to maintain the joint space, thus facilitating the revision surgery [37].

## 5. Method for Mixing the Cement and the Antibiotic

The method of mixing is considered one of the most important factors that affect the release of the antibiotics and the mechanical properties of cement. The preparation should be as porous as possible in order to increase the spread of the antibiotic, but not excessively porous to weaken the structure of the cement itself. A fundamental distinction regards the *method of addition* of the antibiotic to the cement: *manually mixing* at the time of implantation or *industrial mixing* by the several companies which provide premixed antibiotic loaded bone cement [31, 38, 39]. 

The antibiotic must be a powder preparation for a better integration with the cement and a reduced interference with the mechanical properties of the cement [39]. Until now, no studies were conducted to correlate the changes in the release of the antibiotic with the temperature. It must be considered, however, that the process of polymerization of the cement is an exothermic reaction with temperatures up to 60°–80°C. Therefore, the antibiotics destined to be mixed with the cement must be chemically and thermally stable [40]. The manual preparation, according to a study conducted on the Simplex-P spiked with tobramycin, reduces the strength of cement of 36% compared to the ALBC prepared industrially [30]. The improvement of mechanical properties due to the greater compactness of the structure of the cement, however, could lead to a decrease in the rate of diffusion of the antibiotic [32]. This difference however is not considered significant by most surgeons.

## 6. The Choice of the Antibiotic

The choice of the antibiotic is a fundamental issue. The antibiotic must have a broad antibacterial spectrum (including gram positive and gram negative bacteria) and a low percentage of resistant species. The most commonly mixed antibiotics are gentamicin and tobramycin (aminoglycosides with particular effectiveness against gram-negative bacteria) and vancomycin (glycopeptide active mainly on gram-positive like, e.g., *Staphylococcus aureus*). In addition, the antibiotic must provide a local concentration able to overcome the “break point sensitivity limit” of pathogens. This is generally defined as the antibiotic concentration that marks the transition from bacterial sensitivity to induction to resistance to antibiotics for at least three or four weeks. The final aim is to reach appropriate antibiotic concentrations in the tissues and bone avoiding the toxicity of the concentration of the drugs [22].

A study showed that coagulase negative staphylococci are found in the 88% of the infections in patients undergoing a primary arthroplasty where cement loaded with gentamicin was used [41]. A study [42] *in vitro* analyzed the behaviour of *Staphylococcus aureus* in function of the kinetics of gentamicin release in different cements: none of the antibiotic loaded cements was able to immediately reduce the growth of bacteria, but everyone led to a significant decrease in bacterial growth if compared with non-antibiotic cements. Apparently, the CMW3 cement showed the ability to reduce bacterial colonization for a longer period (24–72 hours) compared to other cements. It was also noted that the gentamicin may act differently when added to different cements, although the mechanism of bacterial adhesion is always the same. For example, it was demonstrated that the release of gentamicin is much more effective in Palacos than in Simplex [14]. Similarly, changes in elution related to the type of cement were found also for the vancomycin. The release of this antibiotic was compared in three different types of cement (CMW1, Palacos-R, and Simplex-P) and the first showed an increased release compared with the other two [43]. Other studies on various cements (Cemex, Palacos, and Simplex) demonstrated that vancomycin alone had a minor and less effective release compared with gentamicin [44]. This lower diffusion of the vancomycin would be related to several factors such as physical-chemical properties of the antibiotic, the molecular weight, the stability of the molecules in presence of biological fluids, the temperature, as well as the different morphology of the cement itself (porosity, roughness, surface, etc.). The type of cement and the method of preparation may modify the elution of the antibiotic, although other studies affirm the opposite and argue that the spread of vancomycin and tobramycin would not depend on the type of cement used [45]. 

One study [40] showed that the tobramycin added to the Simplex cement has good activity against 98% of the bacterial tested, using a wide spectrum of pathogens clinically relevant in orthopaedic infections (aerobic Gram-positive and Gram-negative bacteria, anaerobic ones, and mycobacterium). Tobramycin resistant bacteria at the usual systemic concentrations, such as Enterococcus faecalis, methicillin-resistant staphylococci, and Staphylococcus epidermidis, exhibited a limited sensitivity also to the antibiotic released from the cement. The study confirmed the effectiveness of the tobramycin bounded to PMMA for the prevention and reduction of infections caused by a wide spectrum of micro-organisms: tobramycin is stable during the exothermic cement polymerization and its release on the surface of PMMA occurs at concentrations that usually inhibits the growth of the majority of the examined bacteria. Another study from the same authors [46] compared the two principal aminoglycosides used in prosthetic surgery, gentamicin and tobramycin, respectively, added to Palacos and Simplex. The results showed that Simplex-tobramycin has antibacterial activity against 98% of the tested strains of P. Aeruginosa, while Palacos-gentamicin contrasts the 93% of the same bacteria. These results would suggest an antibacterial activity of the tobramycin from 2 to 8 times better than gentamicin. Aminoglycosides act through a mechanism directly correlated to the concentration; therefore increasing the dose of the antibiotics corresponds to an increased antibacterial efficacy. In addition, the release of antibiotic is positively correlated to the quantity added to the cement [47].

## 7. Dose of Antibiotic

The dosage of the antibiotic varies according to the use for which the cement is destined. Many authors argue that in case of acute infections high doses of antibiotics should be used: more than 2 g each 40 g of cement, usually from 6 to 8 g each 40 g, for a prolonged and effective release against pathogens [16, 22, 48]. Whereas if the ALBC is used for prophylaxis in first implants, where the first function of the cement is to fix the implant, the antibiotic can be mixed at low doses: less than 2 g each 40 g of antibiotic cement. An inadequate dose may be seen as the cause of failure of the prosthesis, as it may generate the emergence of resistant bacteria [16, 22, 48].

## 8. Association of More Than One Antibiotic into the Bone Cement

The activity of the release of two or more antibiotics from the bone cement was studied. The idea to add more than one single antibiotic arose after the emergence of resistant bacteria and after the possible synergistic combination of two antibiotics has become an increasingly common practice in infectivology (usually, vancomycin and aminoglycosides are often combined for their synergic potential effect in the treatment of serious infections caused by the *S. aureus*). Since 1970, it has been documented that *β*-lactamic antibiotics can be combined with most of aminoglycosides, when there is a high concentration of both substances, or when their excretion is delayed. The combination of a molecule of a *β*-lactam with an aminoglycoside molecule can inactivate equimolar amounts of both antibiotics. When high doses of both substances are combined with cement, the inactivation of these can affect the properties of the combination, but this phenomenon has not been studied yet [41].

A study conducted on 20 patients with infections due to *S. aureus*, *S. epidermidis*, *E. coli* and *P. aeruginosa*, showed a superior effectiveness of spacers loaded with a combination of gentamicin and vancomycin compared to spacers loaded with gentamicin alone [36]. The emerging capacity of staphylococcal survival on prosthetic materials and the *in vitro *effects of gentamicin and vancomycin-loaded polymethylmethacrylate (PMMA) were studied on hospital acquired staphylococcal strains systematically inoculated on four orthopedic materials: Ultra-High Molecular Weight Polyethylene (UHMPWE), Palamed cement without antibiotics, Palamed-G cement, and Palamed-G cement loaded with vancomycin (1 g of antibiotic each 40 g of cement) [49]. The sample with the association of vancomycin-gentamicin was the most effectively protected from bacterial colonization. The result is coherent with other similar tests carried out by other authors on other cements (Palamed and Palacos) and various antibiotics on other strains of bacteria [50, 51].

The additive or synergistic effect of tobramycin on vancomycin released from acrylic cement was demonstrated [52]. This phenomenon was called “passive opportunism” because the second antibiotic appears to act simply as a soluble passive additive. The elution of tobramycin and of vancomycin alone and combined from the disks of acrylic cement was studied: it was demonstrated that combining two antibiotics in bone-cement improves elution of both antibiotics *in vitro* and may translate into enhanced elution *in vivo* [53].

The characteristics of elution of vancomycin and tobramycin alone and together were compared in two types of cement, Palacos and Simplex [15], divided into three groups: a first group (low) contained 1.2 g of tobramycin and 1 g of vancomycin, a second group (medium) 2.4 g of tobramycin and 2 g of vancomycin, and a third group (high) 3.6 g of tobramycin and 3 g of vancomycin. At low dose both antibiotics showed very low elution as well as Simplex in the medium-dose group. Palacos resulted in a greater release than Simplex in medium- and high- dose groups. In particular, Palacos with high concentration of antibiotics showed a level of activity that passed for more than eighty days the level of minimum inhibitory concentration (MIC) of the most common pathogens [15]. Also, the amount of tobramycin released from Palacos was higher than that of Simplex (10 days). Considering the vancomycin, the kinetics of elution was inadequate in all three groups for both cements (taking as limit the detection of 25 *μ*g/mL). Nevertheless, vancomycin resulted active for the first day. Considering the tobramycin, for groups with low and medium dose an inadequacy in the kinetics of release was observed again, but for the high-dose group (especially for Palacos) the duration of release was high. In general, Palacos has a much higher release level (above the MIC for most common pathogens encountered) and for a longer period of time compared to Simplex and tobramycin showed an improved efficacy profile compared to vancomycin [53, 54]. However, the combination of the two antibiotics greatly increases the release and, probability, the efficacy [36]. In another study about the combination of antibiotics [38] the release of antibiotics from a spacer *in vitro* was measured with the purpose to establish the best pairing cement-antibiotic against specific bacteria (*S. aureus*, *S. epidermidis*, *Enterococcus faecalis*, and MRSA). Palacos-R and three different antibiotics (gentamicin, vancomycin, and teicoplanin) were used alone or in combination (gentamicin plus vancomycin or teicoplanin plus gentamicin). The study showed that the combination of two antibiotics in a spacer has a bactericidal activity more prolonged than a spacer loaded with a single antibiotic. Also, the synergistic action of gentamicin and teicoplanin had superior bactericidal activity compared to gentamicin and vancomycin and the coupling of a glycopeptide with an aminoglycoside covers both Gram-negative and Gram-positive bacteria.

## 9. Factors Influencing the Release of Antibiotic from the Cement and the Role of Surface

According to some authors the release of the antibiotic can last for many days [15], while for the majority of the authors the process occurs for the first days only [55]. Others sustain that it is a process of the duration of few hours [56]. The amount and the duration of the release of the antibiotic from the cement is a debated issue which still has not been completely understood [3, 22, 30, 55, 56].

The release of the antibiotic from the cement is influenced by the type (viscosity) of the cement, by the surface of contact/exchange, by the conditions of the compound, and the type and amount of antibiotic. The antibiotic is released from the surface of the cement and from cracks and voids in the cement itself [57]. The nature of the polymer allows the passage of fluids, allowing the release of the incorporated antibiotic. Nevertheless, while the hydrophobicity of the cement limits this release at less than the 10%, the most of the antibiotic is released in the first hours and days after surgery [58]. In addition, a significant amount may still be trapped in cement for long time [58]. According to a study [59], the Palamed, given the same procedure of preparation, is the cement that permits the biggest release of antibiotic over time (17%), compared with Palacos (8.4%) and CMW (4–5.3%). Many authors interpreted this release as a phenomenon of surface, while others argue what occurs throughout the polymeric matrix. It was shown that the initial release is directly proportional to the roughness of the surface: the higher the roughness, the wider the area of release [36, 48]. Also, a linear correspondence after a week between the porosity of the cement and the release of the antibiotic was demonstrated: the continuous release after several days would depend on the deep penetration of the antibiotic in the cement previously determined by the porosity [36, 48]. 

The effect of the direct contact with the surface of biomaterials, such as PMMA, on the characteristics of the bacteria and a consequent possible change in the population and bacterial resistance were studied [60]. Also, different types of antibiotics were evaluated for this subject: *β*-lactams, aminoglycosides, macrolides, and others investigating the susceptibility to antibiotics both from bacteria adherent to the cement and from nonadherent bacteria. The contact with the material gave significant differences in terms of growth for all tested antibiotics, with the exception of clindamycin. These data suggest that the characteristics of the surface of the material could be important in the interaction with bacteria and that the bone cement can lead to changes in bacterial adhesion to the biomaterial modifying the antibiotic resistance [60, 61].

## 10. Mechanical Properties of Antibiotic Cement

It was suggested, as mentioned above, that the addition of antibiotics may play a role in weakening the structure and the mechanical properties of the cement. Various cements and antibiotics were compared, to determine which might be the more resistant over the time: studies conducted on Palacos-R, CMW1, and CMW3 with and without the addition of gentamicin or Simplex-P with erythromycin, colistin, or tobramycin did not show significant effects on fatigue resistance in comparison to the respective simple cements [33]. It must be noted that the majority of the studies which demonstrated a theoretical disadvantage of the cement loaded with antibiotics are *in vitro* studies [47]. On the contrary, the majority of the clinical studies reported an increased rate of mechanical failures when high dosages were used in comparison with the ALBC loaded at low dose [47, 49].

## 11. Toxicity

At our knowledge, there are no reports in literature of systemic toxicity related to the use of ALBC. Various researches focused about local toxicity, with particular interest to the function of osteoblasts and osteocytes: event though there are no reports of clinical adverse effects on these cells, some *in vitro* studies raised doubts about this subject. In addition, the concerns are more consistent in case of cement loaded at high doses, where the local levels of antibiotics may exceed 200 *μ*g/mL. In particular, when osteoblasts derived from trabecular bone were exposed to materials containing various concentrations of gentamicin (0 to 100 *μ*g/mL), the activity of alkaline phosphates decreased significantly in all the cultures with gentamicin concentration >100 *μ*g/mL, the incorporation of 3H-thymidine decreases at the same concentration of antibiotic, and the total DNA decreases for concentrations ≥700 *μ*g/mL [62, 63]. A study about the effect of tobramycin (concentrations between 0 and 10,000 *μ*g/mL) on osteoblasts showed that local levels <200 *μ*g/mL have no effect on replication of these cells, whereas at concentrations >400 *μ*g/mL replication decreases, and with 10,000 *μ*g/mL cell death occurs. Also the effects of vancomycin on osteoblasts were studied for concentrations ranging between 0 and 10,000 *μ*g/mL: levels of vancomycin <1.000 *μ*g/mL had little or no effect on replication, but concentrations of 10,000 *μ*g/mL caused the death of the osteoblasts [64]. Vancomycin seems to be less toxic than aminoglycosides at high concentrations and Gentamicin has lower critical concentrations than those of tobramycin, despite they are both aminoglycosides. 

## 12. Conclusions

The majority of the studies demonstrated an antibacterial effectiveness of cements loaded with antibiotics in the treatment of deep infections following hip and knee arthroplasty (onestage and two stages). 

The main problem of these analyses is the minimus time of treatment that allows the antibiotic effects without developing bacterial-resistance. Recent studies *in vitro* showed that the highest concentration of antibiotic released is found in the first two days; in contrast, studies *in vivo* did not reach statistically significant evidence.

The literature demonstrated that the best results are obtained with the association of antibiotic-loaded cement and the systemic antibiotic administration, if possible with targeted testing.

Finally, a significant difference between the intravenous administration of antibiotics and the use of the antibiotics into the cement for prophylactic use in patients with standard risk was not found; so it is not advisable to use antibiotic loaded cement for routine as prophylaxis. 

 In conclusion, it is recommended not to trust excessively in the role of antibiotic loaded bone cement and not to give it therapeutic properties that it does not posses. It is clear how ALBCs are more effective than simple cements, but undoubtedly the “window of effectiveness” cannot be attributed only to antibiotics. Other properties related to the cement itself such as roughness, porosity, technique of preparation, and many patient-related features must be reminded. 

However, it is necessary to underline that ALBC, especially if targeted by a specific antibiogram or integrated with an association of molecules more than a single one, is an important aid in the prevention and in the treatment of prosthetic infections.
